# Different distribution patterns of plasmacytoid dendritic cells in discoid lupus erythematosus and lichen planopilaris demonstrated by CD123 immunostaining^☆^^☆☆^

**DOI:** 10.1016/j.abd.2019.11.005

**Published:** 2020-03-20

**Authors:** Azadeh Rakhshan, Parviz Toossi, Maliheh Amani, Sahar Dadkhahfar, Arash Bagheri Hamidi

**Affiliations:** a Department of Pathology, Shohada-e Tajrish Hospital, Shahid Beheshti University of Medical Sciences, Tehran, Iran; b Skin Research Center, Shahid Beheshti University of Medical Sciences, Shohada-e Tajrish Hospital, Tehran, Iran

**Keywords:** Alopecia, Dendritic cells, Discoid, Lupus erythematosus

## Abstract

**Background:**

Clinical and histological features may overlap between lichen planopilaris-associated and discoid lupus erythematosus-associated scarring alopecia.

**Objectives:**

The aim of this study was to demonstrate the cutaneous infiltration of plasmacytoid dendritic cells and to compare their distribution pattern in discoid lupus erythematosus and lichen planopilaris.

**Methods:**

Twenty-four cases of discoid lupus erythematosus and 30 cases of lichen planopilaris were examined for immunostaining of the CD123 marker. The percentage and distribution pattern of plasmacytoid dendritic cells and the presence of the plasmacytoid dendritic cells clusters were evaluted in the samples.

**Results:**

The number of plasmacytoid dendritic cells was higher in the discoid lupus erythematosus specimens. Aggregations of 10 cells or more (large cluster) were observed in half of the discoid lupus erythematosus specimens and only 2 lichen planopilaris, with 50% sensitivity and 93% specificity for differentiating discoid lupus erythematosus from lichen planopilaris.

**Study limitations:**

Incidence and prevalence of discoid lupus erythematosus-associated scarring alopecia in the scalp are low, so the samples size of our study was small.

**Conclusions:**

We suggest that a plasmacytoid dendritic cells cluster of 10 cells or more is highly specific for distinguishing discoid lupus erythematosus from lichen planopilaris. It also appears that CD123 immunolabeling is valuable in both active and late stages of the disease.

## Introduction

Scarring alopecia (cicatricial alopecia) refers to disorders that are characterized by the irreversible destruction of hair follicles and permanent alopecia.^1^, ^2^, ^3^, ^4^ Scarring alopecia is categorized into primary and secondary. Primary scarring alopecia (cicatricial alopecia) implies disorders that directly damage hair follicles such as lichen planopilaris (LPP), discoid lupus erythematosus (DLE) and dissecting cellulitis. Secondary scarring alopecia is caused by unwanted damage to hair follicles as a result of inflammatory processes such as tinea capitis, sarcoidosis, and radiation dermatites.^2^, ^3^, ^4^ LPP and DLE are the most common causes of scarring alopecia.^5^ Clinically, both LPP and DLE appear similar on the scalp making the diagnosis difficult. Several dermoscopic findings such as follicular plugs, speckled pattern of blue-gray dots and white areas have been reported to be characteristic of DLE and target pattern of blue-gray dots, perifollicular scalling to be characteristic of LPP.^6^ However, overlap of the findings might make the histological evaluation necessary.^5^ Sometimes, histological features may also overlap with each other, especially in pauci-inflammatory late fibrosing stages^5^, ^7^; consequently, establishing a definite diagnosis may be challenging and difficult based on clinical, dermoscopic and histological features alone.^5^ These concerns underscore the need for useful adjunct diagnostic techniques, including direct immunofluorescence^5^ and Immunohistochemistry (IHC).^8^, ^9^

Plasmacytoid dendritic cells (PDCs) are antigen-presenting cells that play an important role in the innate immune response. They are not typically found in the normal skin,^10^ but the presence of these cells in inflammatory diseases, infections, and malignancies is notable.^11^ For example, these cells have been identified in the skin lesions of patients with lupus erythematosus,^12^ lichen planus,^13^ dermatomyositis,^14^ Sjogren syndrome, and rosácea.^15^ Several studies have demonstrated the significant role of PDCs in the pathogenesis of lupus erythematosusby producing type 1 interferon. This role has been recognized via CD123 immunolabeling, as a surface marker of PDCs.^12^, ^15^, ^16^ The presence of these cells and their specific distribution pattern in the tissue may help to distinguish lupus erythematosus from other inflammatory disorders such as LPP.^8^, ^17^, ^18^

Given that a proper diagnosis of inflammatory conditions that lead to the destruction of hair follicles is mandatory for the prevention of permanent scarring, we aimed to perform the current study to evaluate the presence and distribution pattern of PDCsby immunostaining the CD123 marker in patients with LPP and DLE, which are the most common causes of primary scarring alopecia.

## Methods

### Subjects and data collection

This retrospective study was based on available data at the Pathology Department of Shohada-e Tajrish Hospital in the period from January 2014 to April 2018. Only scalp lesions that had both clinical and histopathological criteria for a diagnosis of scarring alopecia consistent with “discoid lupus erythematosus” or “lichen planopilaris” were included. For all the patients, the histopathological diagnosis was based on H&E stained sections and 3 special stainings, including PAS (Periodic Acid Schiff) for the evaluation of basement membrane thickening, alcian blue at pH 2.5 for demonstrating the presence and pattern of dermal mucin deposition and orcein staining for the evaluation of elastic fibers. The histopathological criteria for DLE were comprised of interface dermatitis at the interfollicular epidermis and the perifollicular region, dense perivascular and periadnexal lymphoid infiltration (both superficial and deep dermal), increased dermal mucin deposition, and thickening of the basement membrane. The histological criteria for LPP were perifollicular interface dermatitis at the level of the infundibulum and the isthmus and a lack of involvement at the inferior segment of the hair follicle and interfollicular epidermis. Overall, 24 cases of DLE and 30 cases of LPP were included. All the cases were categorized into 2 stages according to the amount of inflammation and fibrosis: the early/active stage, characterized by lymphocytic infiltration and the absence of fibrosis, and the late stage, which showed destroyed hair follicles and replacement by fibrous tracts and minimal or absent lymphocytic infiltration.^19^, ^20^

### Immunohistochemical staining

Formalin-fixed paraffin-embedded tissue sections from all the cases were immunostained for the CD123 marker, mouse anti-human CD123 (Interleukin-3 Receptor Alpha Chain) monoclonal antibody (Clone 7G3) (Master Diagnostica, Spain). From the paraffin blocks, 4 μm (micrometer) tissue sections were prepared on charged slides and then incubated overnight at 37 °C. The sections were deparaffinized, rehydrated, and exposed to epitope retrieval by heat (boiling tissue in EDTA buffer at pH 9 for 30 min at 95 °C). The process was followed by rinsing in distilled water for 3–5 changes and cooling at room temperature for 20 min. Endogenous peroxidase was blocked using a peroxidase solution for 10 min at room temperature. The primary antibody (which was ready to use) was incubated for 30 min, followed byEnVision for 30 min at 37 °C and rinsing with phosphate-buffered saline and the IHC wash buffer. Thereafter, the antibody was detected by DAB (3,3′-diaminobenzidine) for 2 min and was, then, washed with distilled water. Slide mounting was carried out after hematoxylin counterstaining.

### Scoring of CD123 staining

Three parameters of PDCs were mainly evaluated on IHC-stained sections (Table 1):

(1) The percentage of PDCs (CD 123+), calculated by dividing the PDC count by the mononuclear cell count at 3 separate regions that had the most prominent infiltration in serial sections. Then, the PDC content was defined as score 1 (PDC < 10%), score 2 (10% ≤ PDC < 20%), and score 3 (PDC ≥ 20%) (18).
(2) The predominant anatomic location of the PDC infiltrate, including the perifollicular, perivascular, perieccrine, subcutaneous, intrafollicular, dermoepidermal junction, and intraepidermal regions.
(3) The presence of the PDC cluster, defined as the aggregation of at least 5 PDCs. Aggregations of 5–10 PDCs were considered as small clusters and aggregations of more than 10 PDCs were considered as large clusters.^17^, ^18^ Additionally, the anatomic location of small and large clusters was determined according to the locations described above.

### Statistical analysis

The percentage of PDCs was compared between the 2 groups using the independent sample *t*-test. The distribution patterns of PDCs were compared between DLE and LPP using the Fisher exact test. The presence of clusters was compared between the 2 groups using the *χ*^2^ test. A *p*-value of less than 0.05 was considered statistically significant.

*All procedures performed in this study involving human participants were in accordance with the ethical standards of our institutional research committee and with the 1964 Helsinki declaration and its later amendments or comparable ethical standards.

This study was approved by our institutional research committee with no. “IR.SBMU.SRC.REC.1395.43”.

## Results

### Baseline characteristics

In total, 54 IHC-stained slides were examined. DLE specimens were taken from 24 patients (7 males and 17 females) at a mean age of 45.33 ± 12.47 years, and LPP specimens were taken from 30 patients (12 males and 18 females) at a mean age of 49.50 ± 13.52 years. There was no significant difference between the 2 groups regarding the age (*p* = 0.245). Nine (37.5%) cases of DLE and 15 (50%) cases of LPP were in the early/active stage of disease, and 15 (62.5%) cases of DLE and 15 (50%) cases of LPP were in the late stage of disease. There was no significant difference between the 2 groups regarding the stage of disease (*p* = 0.3580).

### Percentage and anatomic distribution pattern of PDCs

**Figure 1:**
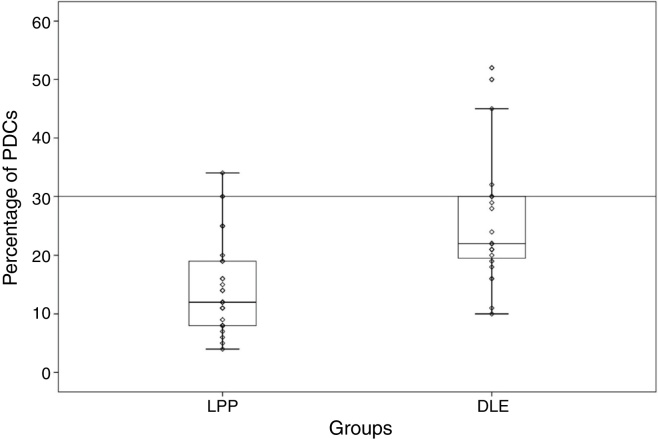
Percentage of plasmacytoid dendritic cells in discoid lupus erythematosus (DLE) and lichen planopilaris (LPP). Middle point, median; Box, interquartile range; Whisker, range (excluding outliers).

The mean percentage of PDCs was 14.87 ± 7.99 (range: 4%–34%) in the LPP cases and 25.45 ± 10.78 (range: 10%–52%) in the DLE cases, which showed a significant difference between the 2 groups (*p* < 0.001) (Fig. 1). In the DLE cases, 0/24 (0%), 6/24 (25%), and 18/24 (75%) of the specimens were scored 1, 2 and 3, respectively. For the LPP cases, 9/30 (30%), 14/30 (46.6%), and 7/30 (23.3%) of the specimens were scored 1, 2 and 3, correspondingly, which was statistically significant (*p* < 0.001) (Table 1).

**Table 1:** Comparisons of the percentages, cluster formation, and anatomic location of PDCs between DLE and LPP.

	DLE	LPP	*p*-Value
*Mean percentage of PDCs*	25.45 ± 10.78	14.87 ± 7.99	<0.001

*PDC score*			
Score 1 (<10%)	0	9 (30%)	<0.001
Score 2 (10 ≤ PDC < 20%)	6 (25%)	14 (46.6%)	
Score 3 (≥20%)	18 (75%)	7 (23.3%)	

*Cluster formation*			
Small cluster	4 (16.7%)	10 (33.3%)	0.002
Large cluster	12 (50%)	2 (6.7%)	

*Anatomic location of PDCs*			
Epidermal	0	0	–
DEJ	15 (62.5%)	2 (6.7%)	<0.001
Interstitial	2 (8.3%)	14 (46.7%)	0.020
Intrafollicular	3 (12.5%)	0	0.082
Perifollicular	24 (100%)	30 (100%)	–
Perivascular	22 (91.7%)	29 (96.7%)	0.579
Perieccrine	4 (16.7%)	0	0.034
Subcutaneous	11 (45.8%)	6 (20%)	0.042

Small cluster, aggregations of 5–10 PDCs; large cluster, aggregations of 10 PDCs or more; DLE, discoid lupus erythematosus; LPP, lichen planopilaris; PDCs, plasmacytoid dendritic cells; DEJ, dermoepidermal Junction.

**Figure 2:**
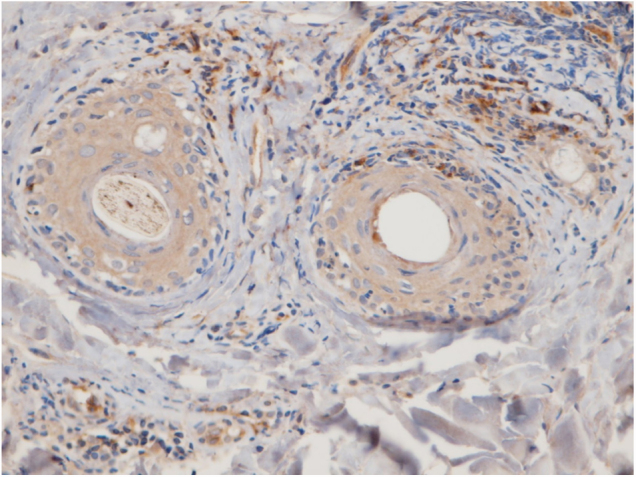
Representative discoid lupus erythematosus case: CD123 immunohistochemistry presence of PDCs at the intrafollicular and perifollicular area (40×).

**Figure 3:**
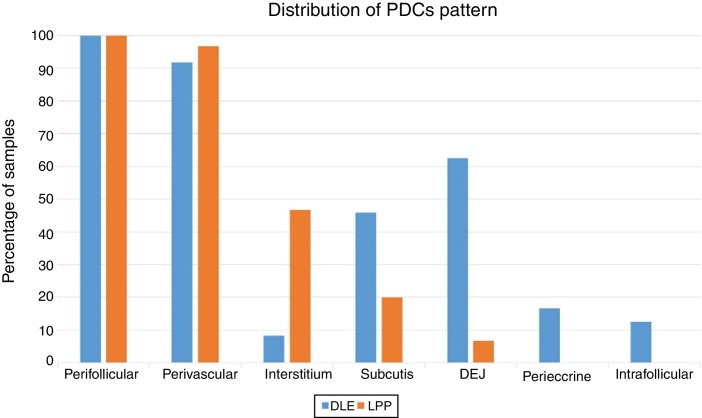
Distribution patterns of plasmacytoid dendritic cells (PDCs) in lichen planopilaris (LPP) and discoid lupus erythematosus (DLE).

The perifollicular region was the most common location involved (all the cases of DLE and LPP had perifollicular infiltrations) (Fig. 2). Perivascular involvement was observed in 29/30 (96.7%) of the LPP cases and 22/24 (91.7%) of the DLE cases, which was not statistically significant (*p* = 0.579). The infiltration of the dermoepidermal junction was detected in 15/24 (62.5%) of the DLE cases and 2/30 (6.7%) of the LPP cases, which was statistically significant (*p* < 0.001). Subcutaneous involvement was identified in 11/24 (45.8%) of the DLE cases and 6/30 (20%) of the LPP cases; this differentiation was statistically significant (*p* = 0.042). Perieccrine infiltration was observed in 4/24 (16.7%) of the DLE cases and 0/30 of the LPP cases, showing statistical significance (*p* = 0.034). Interstitial infiltration was observed in 14/30 (46.7%) of the LPP cases and 2/24 (8.3%) of the DLE cases, which was also statistically significant (*p* = 0.02). Intrafollicular involvement was detected in 3/24 (12.5%) of the DLE cases and 0/30 of the LPP cases (Fig. 2), which was not statistically significant. Intraepidermal infiltration of PDCs was observed neither in the DLE cases nor in the LPP cases (Fig. 3 shows the anatomical distribution pattern of PDCs).

### PDC cluster formation

**Figure 4:**
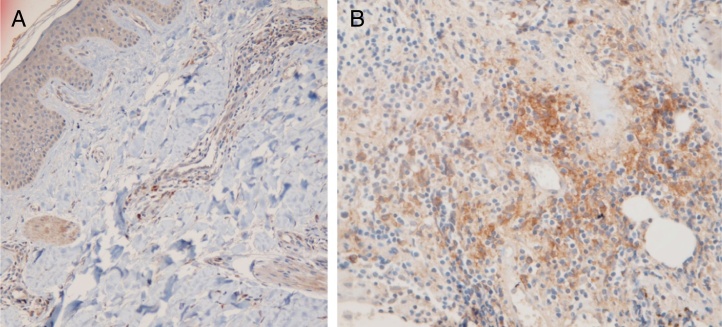
Representative lichen planopilaris: presence of PDCs (no cluster formation), 20× (A) and representative discoid lupus erythematosus case: large cluster (aggregation of 10 cells or more) of plasmacytoid dendritic cells, 40× (B).

Fifteen small clusters (aggregation of 5–10 cells) were identified. Ten of these small clusters were observed in 10 cases of LPP (33.33%) and 5 of them were observed in 4 cases of DLE (16.7%). The location of these clusters in both LPP and DLE was perifollicular and perivascular. Twenty-six large clusters (aggregation of 10 cells or more) were identified. Three of these large clusters were detected in 2 (6.7%) cases of LPP and 23 of them were identified in 12 (50%) cases of DLE. There was a significant difference between these 2 groups regarding cluster formation (*p* = 0.002). The presence of large clusters had 50% sensitivity and 94% specificity for the differentiation of DLE from LPP (Fig. 4A and B).

The anatomic sites of the large clusters were also identified. The large clusters in the DLE specimens were often observed at the perifollicular region (21/23 of the clusters). One large cluster was observed at the dermal–epidermal junction region, and another one was observed at the subcutis. In the LPP specimens, the large clusters were observed at the perifollicular (1), perivascular (1), and interstitial (1) regions.

Half of the LPP cases with small clusters were in the early/active stage of disease and the other half were in the late stage of disease, which was not statistically significant (*p* = 1.000). Of all the DLE cases with small clusters, one case had active disease and 3 cases were at the late stage of disease, which was not statistically significant (*p* = 1.000). Two cases of LPP had large clusters: 1 in the active stage of disease and the other one in the late stage of disease, which was not statistically significant (*p* = 1.000). Of the 12 cases of DLE with large clusters, 4 cases had active disease and 8 cases were at the late stage of disease, which was not statistically significant (*p* = 0.673).

## Discussion

Lupus erythematosus is an autoimmune disease that involves multiple organs. PDCs and type 1 interferons have an important role in the pathogenesis of lupus erythematosus. Studies have demonstrated that PDCs migrate from blood to the site of inflammation such as the skin and the kidney in lupus erythematosus.^16^ Large numbers and aggregations of PDCs have been described in many types of cutaneous lupus (e.g. acute lupus and DLE). PDCs have been shown to have greater numbers in cutaneous lupus erythematosus than in other inflammatory skin diseases such as dermatomyositis, rosacea, and lichen planus.^12^, ^14^, ^15^, ^21^ Our study showed a high percentage of PDCs in scarring alopecia due to DLE by comparison with LPP (approximately 26% vs. 15%).

PDCs have been demonstrated in the mucosal and cutaneous lesions of lichen planus.^22^, ^23^ The number of these cells varies in the different studies having been conducted hitherto. In some studies, a low percentage of PDCs has been reported in LPP (<10%) (8, 17, 18), whereas a higher percentage of PDCs in oral lichen planus (20%) has been shown.^23^ In our study, the percentage of PDCs in LPP was approximately 15%, which is higher than the figure in the studies by Fening and Seleimans.^8^, ^18^

**Figure 5:**
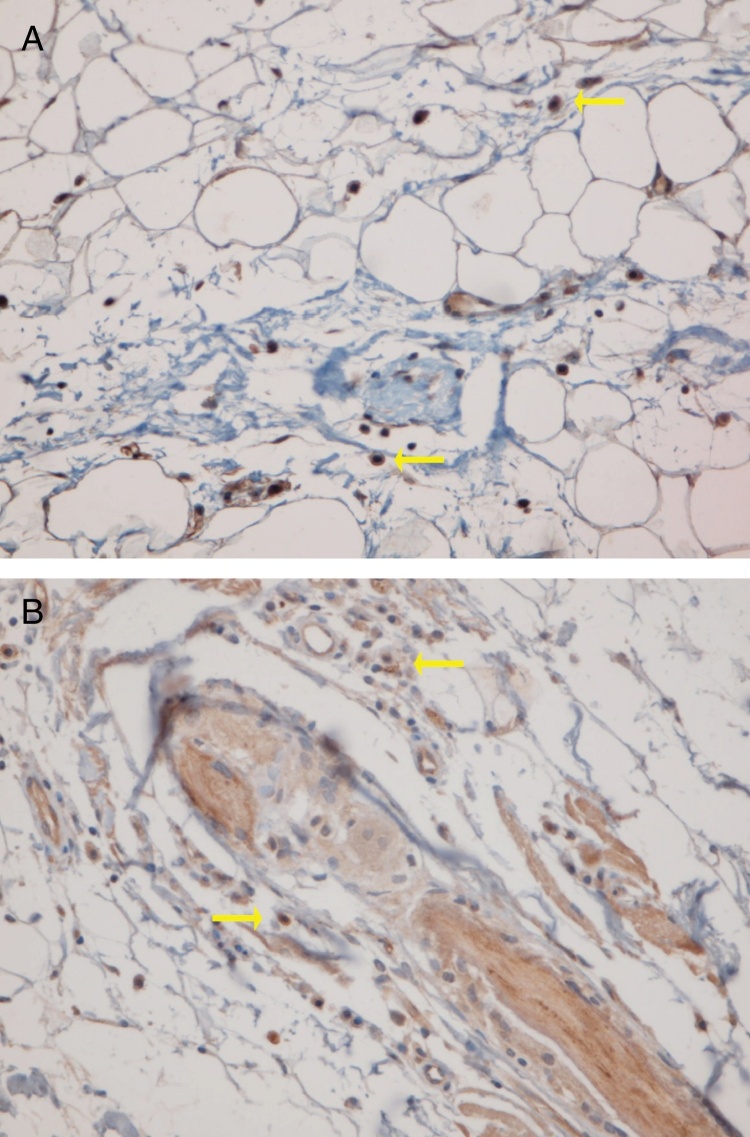
Presence of plasmacytoid dendritic cells scattered through the subcutis in a case of discoid lupus erythematosus, 40× (A) and mostly around a follicular stela at the subcutis in a case of lichen planopilaris, 40× (B).

The predominant anatomic site of PDC infiltration can also vary in the skin tissues of both DLE and LPP. Sleiman et al. reported the presence of PDCs in deep dermis, perieccrine, and dermoepidermal junction areas in almost all DLE specimens, while the infiltration of these cells at the dermoepidermal junction was observed in only a small number of LPP specimens and deep dermis and the perieccrine region were not involved in LPP specimens.^18^ In the study of Fening et al., perifollicular and perivascular involvement was observed in almost all DLE specimens and small numbers of LPP specimens, as well as perieccrine and perifollicular involvement, were observed in many DLE specimens but not in LPP specimens. In their study, the intrafollicular involvement of PDCs was observed neither in DLE nor in LPP.^8^ In our study, the perifollicular and perivascular regions showed the highest density of PDCs in both DLE and LPP. Vertical histopathological sections in our study enabled the evaluation of epidermis and the dermoepidermal junction, which cannot be assessed when the sections are horizontal. Neither DLE nor LPP showed the intraepidermal infiltration of PDCs. The involvement of the dermal–epidermal junction and the subcutaneous region was notable in our DLE specimens. We detected perieccrine and intrafollicular infiltration in a small number of the DLE cases but not in any of the LPP cases (Fig. 2). Interstitial infiltration was observed in many of the LPP cases and small number of the DLE cases. Our study is the first study of its kind to evaluate the subcutaneous region for the presence of PDCs in DLE and LPP, and we detected these cells at the subcutaneous tissue in both DLE and LPP. It should be noted that PDCs were mostly observed along the deep part of hair follicles at the subcutaneous tissue of the LPP specimens, while they were more scattered through the subcutis in the DLE specimens (Fig. 5).

In our study, we observed large clusters (aggregations of 10 cells or more) in half of the DLE specimens but in only 2 cases of LPP which had 50% sensitivity and 94% specificity for differentiation of DLE from LPP. These large clusters were often localized at the perifollicular region. Theodore et al. reported aggregations of 20 cells or more in 60% of facial lupus specimens.^15^ Sleiman et al. detected aggregations of 10 cells or more in 100% of DLE cases with scarring alopecia.^18^ Other studies have detected a few or no clusters of PDCs in other causes of scarring alopecia such as LPP, frontal fibrosing alopecia, and central centrifugal cicatricial alopecia.^8^, ^17^, ^18^ Koliver et al. estimated that clusters of 5 cells or more had 77% sensitivity and 89% specificity to distinguish DLE from LPP.^17^ In our study, the small clusters (aggregation of 5–10 cells) did not have any diagnostic value in differentiating LPP from DLE.

Fening et al. excluded cases that did not have significant inflammation of mononuclear cells and then identified aggregations of 10 PDCs or more and 20 PDCs or more in 94.1% and 82.4% of cases of DLE with scarring alopecia, respectively.^8^ According to our study, cluster formation can be seen in both active (prominent inflammation without fibrosis) and late stages (prominent fibrosis with minimal or without inflammation) of disease.

## Conclusion

Based on our study, it appears that the detection of PDCs by immunostaining the CD123 marker is an acceptable diagnostic method for the diagnosis of DLE and the presence of clusters of 10 cells or more has high specificity for the diagnosis of DLE-associated scarring alopecia. It also appears that CD123 immunolabeling is valuable in both active and late stages of disease.

## Financial support

None declared.

## Authors' contributions

Azadeh Rakhshan: Elaboration and writing of the manuscript; obtaining, analysis, and interpretation of the data; critical review of the manuscript.

Parviz Toossi: Approval of the final version of the manuscript; conception and planning of the study; critical review of the manuscript.

Maliheh Amani: Statistic analysis; conception and planning of the study; elaboration and writing of the manuscript; obtaining, analysis, and interpretation of the data; effective participation in research orientation.

Sahar Dadkhahfar: Obtaining, analysis, and interpretation of the data; critical review of the manuscript.

Arash Bagheri Hamidi: Statistic analysis; obtaining, analysis, and interpretation of the data.

## Conflicts of interest

None declared.
